# Lung Volume Change Assessment in Moderate and Severe COVID-19 Using CT Volumetry

**DOI:** 10.3390/diagnostics15121465

**Published:** 2025-06-09

**Authors:** Alin Iulian Feiereisz, George-Călin Oprinca, Victoria Birlutiu

**Affiliations:** 1 Faculty of Medicine, Lucian Blaga University of Sibiu, Str. Lucian Blaga, Nr. 2A, 550169 Sibiu, Romania; georgecalin.oprinca@ulbsibiu.ro (G.-C.O.); victoria.birlutiu@ulbsibiu.ro (V.B.); 2 Infectious Diseases Clinic, Academic Emergency Hospital Sibiu, 550245 Sibiu, Romania; 3 Department of Pathology, Academic Emergency Hospital Sibiu, 550245 Sibiu, Romania

**Keywords:** COVID-19, CT volumetry, respiratory failure, alveolar collapse, affected lung, 3D Slicer

## Abstract

**Background/Objectives: Background**: COVID-19 pneumonia leads to alveolar collapse and parenchymal infiltration, contributing to lung volume loss and respiratory failure. **Objectives**: To quantify lung volume loss and recovery in moderate and severe cases, explore mechanisms of respiratory failure, and correlate imaging findings with histopathological changes. **Methods**: We retrospectively analyzed 43 patients with moderate/severe COVID-19. CT scans from the acute phase and at 3–12 months follow-ups were processed using 3D Slicer. Infiltrated (−650 to −200 HU) and collapsed (−200 to 0 HU) lung regions were quantified and summed to define the affected lung volume. CT severity scores and total affected percentage were compared with lung volume loss. Histopathological analysis of three autopsy cases was used to support imaging findings. **Results**: Median acute phase lung volume loss was 30.6%. Patients with <25%, 25–50%, and >50% affected lung had median losses of 6.5%, 35.7%, and 39.8%, respectively. Volume loss strongly correlated with affected lung percentage (r = 0.72, *p* < 0.000001) and moderately with CT severity score (r = 0.52, *p* < 0.01). Histology confirmed alveolar area reductions over 65% in infiltrated regions. **Conclusions**: Lung volume loss reflects both imaging severity and histopathological damage, offering insights into the mechanisms of COVID-19 respiratory failure. CT volumetry is a valuable tool for assessing parenchymal injury and monitoring recovery, and 3D Slicer provides an accessible platform for implementing this approach.

## 1. Introduction

Lung injuries in COVID-19 result from a series of pathological events that damage the pulmonary parenchyma and blood vessels, ultimately leading to the loss of the ability to ensure gas exchange. The entry of the SARS-CoV-2 virus into the pulmonary epithelium and its replication within these cells leads to viral recognition by antigen-presenting cells. These cells process the viral particles and present them to natural killer (NK) cells and cytotoxic T cells (CD8+) [1]. The activation of innate and adaptive immunity will be accompanied by the release of pro-inflammatory cytokines and chemokines, such as interleukin (IL)-1β, IL-6, and tumor necrosis factor alpha (TNF-α), ICAM1, CXCL12. Molecules are able to recruit macrophages and neutrophils which, in response, release oxygen radicals (ROS) and proteases with the effect of damaging the alveolar epithelium [2].

The recruitment of macrophages, neutrophils, and monocytes will be associated with alveolar, endothelial, and vascular damage through the renin–angiotensin–aldosterone system (RAAS) by inhibiting the conversion of angiotensin (Ang) II to Ang 1–7, leading to increased plasma levels of Ang II and the induction of pro-inflammatory cytokine synthesis via NF-κB. This effect also extends to the surrounding healthy tissue areas, activating the macrophages present at this level causing a mild inflammatory response [3,4].

Viral invasion occurs through the binding of the Spike protein to the angiotensin-converting enzyme 2 (ACE2) receptor or through the activation by the transmembrane serine protease 2 (TMPRSS2), and subsequent inflammation leads to edema as a result of increased vascular permeability with the presence of rich transudate in the intravascular space—in the alveolar interstitium. This disrupts lung micromechanics and decreases alveolar elastic properties, leading to reduced alveolar volume. The fluid accumulation also reduces the surface area available for gas exchange and impairs the expansion capacity of the lungs by altering lung compliance, ultimately resulting in a reduction in total lung volume [5].

The SARS-COV-2 virus damages type I and II pneumocytes and the endothelial cells of the pulmonary capillary vessels both directly and through the effect of the inflammatory response. Type I pneumocytes are involved in gas exchange ensuring a large surface necessary for easy diffusion. Type II pneumocytes, on the other hand, are involved in the production of a surfactant, an important agent in preventing alveolar collapse during breathing. The destruction of these cells reduces the gas exchange capacity and modifies the production and properties of the surfactant—factors that lead to the loss of alveolar stability and contribute to the collapse of certain areas [6].

Another mechanism that contributes to the reduction in volumes is the appearance of early fibrosis in the acute phase, although extensive fibrosis is a characteristic of chronic lung diseases. Fibrin accumulates as a response to inflammation in the alveolar space and at the level of the alveolar septa, which stiffens the pulmonary test, changing its physiological-mechanical properties, resulting in a decrease in the expansion of the affected test [7].

We can also observe changes in the distribution of collagen, one of the proteins with a structural role in the lung tissue that contributes to their resistance and flexibility. Proteoglycans and their negatively charged glycosaminoglycan (GAG) chains surround the collagen fibrils in a gel-like matrix. The extracellular matrix (ECM), a complex network, is crucial for maintaining mechanics and total lung volume during respiration. Pathogenetic changes at the pulmonary level identified by other authors relate to the reduction in the four surfactant proteins, with the loss of surfactant in cases of respiratory distress, skeletal changes, polarity changes, and cellular functions of cell–matrix adhesion, loss of ECM (such as elastin, glycoproteins, proteoglycans, fibrillar collagens) of the basal membrane [8].

In healthy lungs, negatively charged GAG chains repel one another, which allows collagen fibers to slide and stretch during respiration, increasing lung compliance and total lung volume. Inflammation disturbs this delicate balance and the inflammatory mediators IL-1β and TNF-α can stimulate the MMP enzymes that break down the GAG chains of proteoglycans, resulting in the reduction in the space between the collagen fibers and the ability to slide and stretch [9].

The combined effects of airspace collapse, cellular damage, surfactant dysfunction, early-stage fibrosis, and the distribution of collagen and fibrin significantly reduce the surface area available for gas exchange as well as the mechanical properties of the lungs. Additionally, coagulation disorders contribute to the condition, with hypercoagulability causing microthromboses that exacerbate oxygen deficiency. This coagulation dysfunction also impairs the ability to regenerate and repair blood vessels. The progression of sepsis, along with increased production of pro-inflammatory cytokines, is associated with consumptive coagulopathy and the activation of specific immune signaling pathways, such as NF-κB/NFKB2 or Fc-epsilon, in the lungs—features that are unique to SARS-CoV-2 pathology. Extensive remodeling of lesions in the alveolar and vascular regions is associated with pulmonary fibrosis and pulmonary hypertension [10].

## 2. Materials and Methods

### 2.1. Setting and Participants

We conducted a retrospective observational study including 43 adult patients with moderate or severe COVID-19 pneumonia confirmed by RT-PCR. All patients underwent chest CT during the acute phase due to respiratory symptoms and had at least one follow-up CT scan performed 3 to 12 months after diagnosis. The study was conducted at the Academic Emergency Hospital Sibiu, Romania. Patients were selected based on the availability of high-resolution CT scans in the DICOM format and appropriate clinical documentation.

### 2.2. Imaging Post-Processing

**Figure 1:**
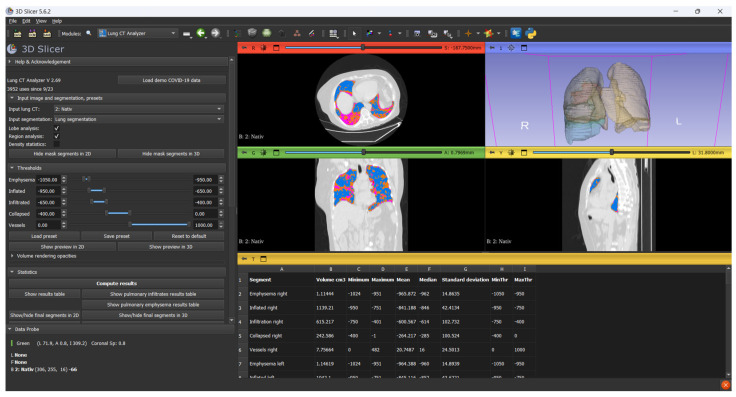
3D Slicer software-Chest Imaging Platform.

Before image processing, all CT scans in the DICOM format were anonymized using DicomCleaner (Radiological Society of North America, open-source) to remove all patient-identifiable metadata. Each scan was assigned a unique numerical Case ID to ensure secure tracking throughout the analysis pipeline. Only anonymized, de-identified datasets were imported into 3D Slicer (version 5.6.2; https://www.slicer.org, accessed on 4 February 2025) for further image processing (Figure 1).

Lung segmentation and volumetric analysis were performed using modules from the Chest Imaging Platform. The segmentation process was initiated with Lung CT Segmenter, which automatically delineates the lung parenchyma. Subsequent classification and volumetric quantification were carried out using Lung CT Analyzer (version 2.69).

A threshold-based classification approach was applied based on standard Hounsfield Unit (HU) ranges: emphysematous regions were defined between −1050 and −950 HU, normally aerated lung between −950 and −650 HU, infiltrated parenchyma (including ground-glass opacities and consolidation) between −650 and −200 HU, and collapsed regions between −200 and 0 HU. Structures above 0 HU, including vascular and mediastinal components, were excluded.

The affected lung volume was defined as the sum of voxels between −650 and 0 HU, comprising both infiltrated and collapsed regions. These thresholds were selected based on prior studies of viral pneumonia, which demonstrated that high-attenuation areas within this range correlate with radiological involvement and allow reproducible quantification of disease extent [11,12].

The software provided separate volume measurements corresponding to each predefined radiodensity interval, allowing structured analysis of both aerated and non-aerated lung tissue. This enabled a precise evaluation of emphysematous, normally aerated, infiltrated, and collapsed lung regions. The affected lung volume was defined as the sum of infiltrated and collapsed areas. Volume loss was computed by comparing total lung volume in the acute phase to that measured at the follow-up.

All CT scans were independently scored by two board-certified radiologists with more than five years of experience in thoracic imaging. In cases of discrepancy, the final score was established through consensus review.

Pulmonary involvement on chest CT was assessed using a 25-point severity scoring system based on the protocol originally described by Pan et al., which we adapted to our study population [13]. This semi-quantitative method evaluates the extent of parenchymal abnormalities in each of the five pulmonary lobes. Each lobe is assigned a score from 0 to 5 according to the estimated percentage of involvement: 0 for no involvement, 1 for less than 5%, 2 for 5–25%, 3 for 26–50%, 4 for 51–75%, and 5 for more than 75% involvement. The total CT severity score (CTSS) is obtained by summing the scores of all five lobes, with a maximum possible value of 25.

### 2.3. Histopathological Analysis

Three full autopsies were performed in the red-zone (restricted COVID-19 area) of the County Clinical Emergency Hospital’s morgue. The patients had died following confirmed severe COVID-19 pneumonia, as verified by rt-qPCR analysis of nasopharyngeal swabs collected immediately after admission. All three patients had undergone chest CT examinations at admission, which revealed extensive pulmonary lesions with CT severity scores greater than 20, indicating widespread parenchymal involvement. Following a meticulous macroscopic examination of the internal organs and a detailed description of any anomalies found, tissue samples were collected from multiple areas of the pulmonary parenchyma and then fixed in a 10% formalin solution. After fixation, the tissue samples were embedded in paraffin blocks for the purpose of creating microscopic hematoxylin–eosin slides, as in other research conducted by our laboratory [14].

**Figure 2:**
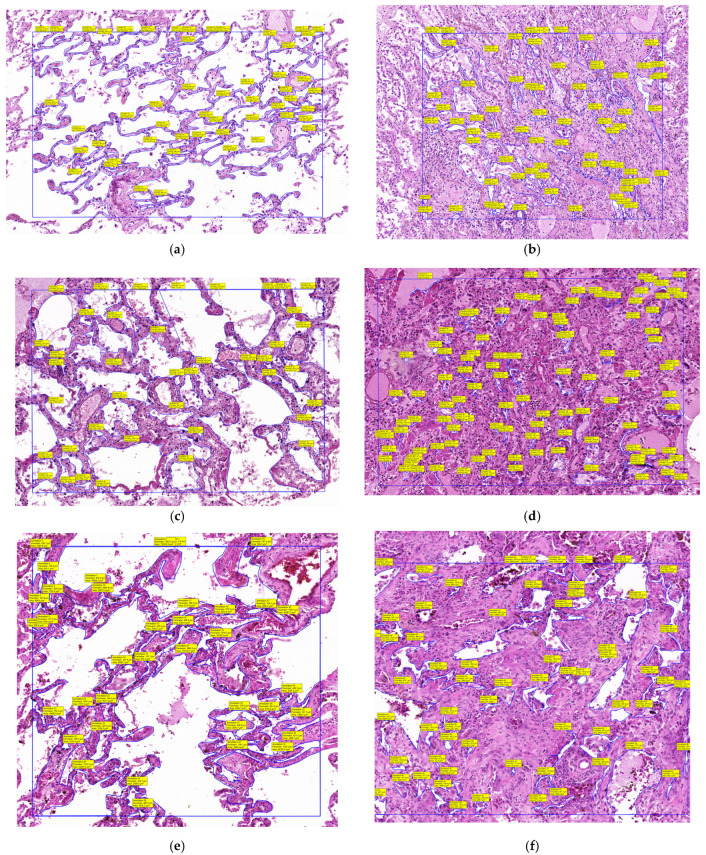
Histopathological analysis. Total area within 1 mm^2^ of unaffected lung tissue and within a 1 mm^2^ area of condensed lung: (**a**) Case 1: unaffected lung; (**b**) Case 1: condensed lung; (**c**) Case 2: unaffected lung; (**d**) Case 2: condensed lung; (**e**) Case 3: unaffected lung; (**f**) Case 3: condensed lung.

The slides were then digitally scanned using the Panoramic Desk II DW digital slide scanner (3DHistech, Budapest, Hungary) and examined with the 3D Histech Slide Viewer application. Once digitized and opened in the Viewer application, all alveolar spaces within a 1 mm^2^ area were manually selected using the closed polygon annotation tool from the Viewer. After the selection process was completed, the tool calculated all selected areas and generated an Excel file with the alveolar space areas within that 1 mm^2^. An average alveolar space area was then calculated. For each patient, we calculated the mean area within 1 mm^2^ of unaffected lung tissue and within a 1 mm^2^ area of condensed lung (Figure 2).

### 2.4. Statistical Analysis

Volumetric and densitometric parameters were collected for all patients at two time points: during the acute phase of COVID-19 pneumonia and at follow-up imaging after recovery (Table 1).

For each CT scan, total lung volume was measured, and the affected lung volume was calculated as the sum of infiltrated regions (−650 to −200 HU) and collapsed regions (−200 to 0 HU), segmented using threshold-based classification. To allow for meaningful inter-patient comparisons, lung volumes were also expressed as percentages, accounting for physiological variability due to sex, height, and body size. Patients were stratified using two complementary severity classification systems. The first was based on the percentage of affected lung volume in the acute phase (<25%, 25–50%, and >50%). The second used the 25-point CT Severity Score (CTSS), following the method described by Pan et al., with patients categorized as mild (<8 points), moderate (9–15 points), or severe (>15 points).

Normality of data distribution was assessed using the Shapiro–Wilk test. For group comparisons, the Kruskal–Wallis test was applied. Post hoc analysis was performed using the Mann–Whitney U test, with Bonferroni correction for multiple comparisons. Correlations between continuous variables were assessed using Spearman’s rank correlation coefficient. All statistical analyses were performed using IBM SPSS Statistics version 26.0.

## 3. Results

### 3.1. Correlation Between Affected Lung and Volume Reduction

In the study cohort (*n* = 43), a strong positive correlation was observed between the percentage of affected lung parenchyma in the acute phase—defined as the combined volume of infiltrated and collapsed regions within the −650 to 0 HU range—and the percentage of total lung volume reduction, calculated by comparing acute-phase and follow-up CT scans. To account for physiological variability in lung size (e.g., sex and height), both parameters were expressed as percentages, which offers the advantage of enabling meaningful comparisons across individuals with different baseline lung capacities.

**Figure 3:**
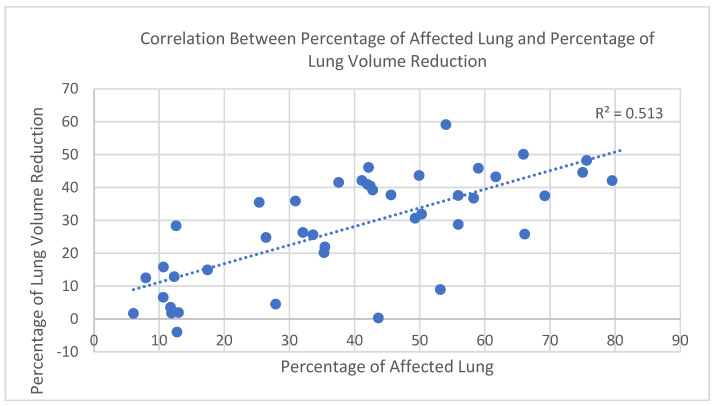
Correlation between percentage of affected lung and percentage of lung volume reduction.

The relationship was assessed using Spearman’s rank correlation coefficient due to the non-normal distribution of volume loss values (confirmed by the Shapiro–Wilk test). The correlation was statistically significant, with r = 0.72 and *p* < 0.000001 (Figure 3), indicating a strong positive association between parenchymal involvement and lung volume reduction during the acute phase.

### 3.2. Lung Volume Reduction by Radiological Severity

Lung volume reduction during the acute phase was compared across severity groups defined by both the percentage of affected lung parenchyma and the CT Severity Score (CTSS).

**Figure 4:**
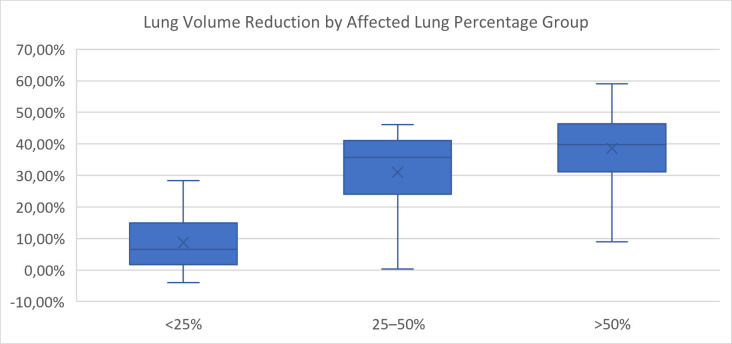
Lung volume reduction by affected lung percentage group.

When grouped by percentage of affected lung, a significant overall difference in volume reduction was observed (*p* < 0.0001, Kruskal–Wallis test) (Figure 4). Post hoc pairwise comparisons using the Mann–Whitney U test with Bonferroni correction (adjusted α = 0.0167) revealed that patients with <25% affected lung had significantly lower volume reduction than those in the 25–50% group (*p* = 0.0005) and the >50% group (*p* = 0.0001). The difference between the 25–50% and >50% groups was not statistically significant (*p* = 0.084).

**Figure 5:**
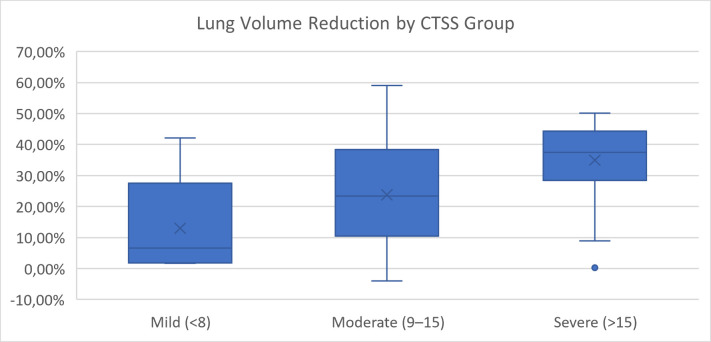
Lung volume reduction by CTSS group.

When patients were grouped by CTSS, a significant overall difference was also detected (*p* = 0.0145, Kruskal–Wallis test) (Figure 5). However, none of the pairwise comparisons between mild, moderate, and severe groups reached statistical significance after Bonferroni correction. The comparisons between mild and severe (*p* = 0.029) and between moderate and severe (*p* = 0.019) approached significance but did not meet the adjusted threshold.

These results suggest that HU-based volumetric classification more clearly distinguishes structural lung volume reduction than semi-quantitative CTSS grouping, particularly in intermediate severity categories.

### 3.3. Absolute Lung Volume Reduction by Radiological Severity

Absolute lung volume reduction between the acute phase and follow-up was compared across radiological severity groups. When stratified by the percentage of affected lung, patients in the <25% group had a median lung volume reduction of 284.4 mL, those in the 25–50% group had 1730.4 mL, and the >50% group showed a median reduction of 1993.4 mL. The difference between groups was statistically significant (*p* < 0.0001, Kruskal–Wallis test). Pairwise comparisons indicated significantly greater volume reduction in both the 25–50% and >50% groups compared to the <25% group (*p* = 0.0006 and *p* = 0.0001, respectively; Bonferroni-adjusted Mann–Whitney U tests), while no significant difference was found between the 25–50% and >50% groups (*p* = 0.39).

Similarly, stratification by CT Severity Score (CTSS) showed a median lung volume reduction of 284.4 mL in the mild group (CTSS < 8), 1114.8 mL in the moderate group (CTSS 9–15), and 1935.7 mL in the severe group (CTSS > 15). The overall difference was statistically significant (*p* = 0.014, Kruskal–Wallis test), although none of the pairwise comparisons between CTSS groups reached significance after Bonferroni correction.

These results suggest that HU-based volumetric classification more clearly distinguishes structural lung volume reduction than semi-quantitative CTSS grouping, particularly in intermediate severity categories.

### 3.4. Histopathological and Radiological Correlation in Severe Cases

To assess whether CT-defined infiltrated and collapsed regions reflect true alveolar damage, three fatal cases of severe COVID-19 pneumonia were analyzed, each presenting with CT Severity Scores exceeding 20 at admission. Volumetric CT revealed markedly reduced total lung volumes, with affected regions accounting for 1766 mL out of 2006 mL, 1931 mL out of 2355 mL, and 1480 mL out of 2408 mL, respectively.

**Figure 6:**
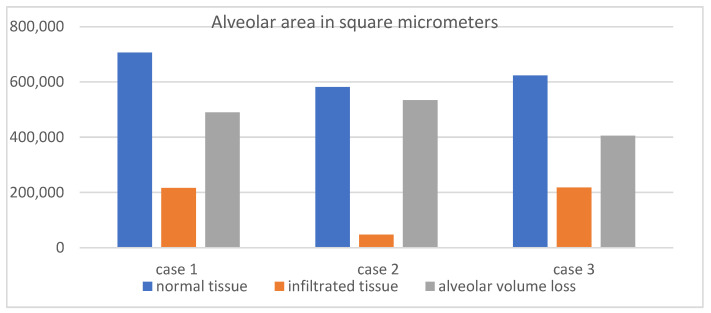
Alveolar area in square micrometers.

Following digital microscopy measurements, a pronounced difference in the alveolar area was observed between normal and affected regions, with the total alveolar area reduced by more than 65% in infiltrated zones. This histological finding supports the volumetric CT results (Table 2, Figure 6), confirming that the radiologically defined abnormalities represent true structural loss of alveolated parenchyma.

## 4. Discussion

In the context of acute respiratory failure, especially in patients with COVID-19 pneumonia, the assessment of lung function is significantly limited by the inability to perform conventional pulmonary function tests (PFTs). Techniques such as spirometry, lung plethysmography, or gas diffusion studies are effort-dependent and generally contraindicated in critically ill patients due to the risk of worsening respiratory compromise, aerosol generation, and poor patient cooperation. In contrast, quantitative CT volumetry provides a robust, non-invasive method to estimate lung volume and aeration status directly from routinely acquired chest CT scans. This approach is grounded in objective radiological principles and has been increasingly validated in published studies as a surrogate for physiological lung function. By applying standardized Hounsfield Unit (HU) thresholds, different lung compartments—such as normally aerated, infiltrated, or collapsed regions—can be segmented and quantified with high reproducibility.

Multiple studies have demonstrated a strong correlation between CT-derived lung volumes and PFT results in stable patients, confirming its validity as a structurally based proxy for functional assessment.

This study investigated lung volume changes in patients with moderate-to-severe COVID-19 using CT volumetry and histopathological analysis, shedding light on the pathophysiology of SARS-CoV-2-induced lung injury and recovery. Our findings provide insights into the mechanisms of respiratory failure and have broader implications for understanding lung injury in other types of pneumonia. Below, we compare our results with the recent literature, discuss limitations, and explore the relevance of our findings beyond COVID-19.

In this study, volumetric lung analysis was performed using 3D Slicer, an open-source medical image computing platform that has become increasingly recognized in clinical research. One of the platform’s major advantages is its accessibility: It is free, highly extensible, and supports a wide range of segmentation tools, including plugins specifically designed for chest CT analysis, such as the Lung CT Segmenter and Lung CT Analyzer extensions. Despite its open-source nature, 3D Slicer has shown comparable performance to commercial software in multiple research settings. Prior studies have demonstrated that HU-based segmentation performed in 3D Slicer yields accurate and reproducible lung volume estimates, aligning closely with measurements from proprietary tools used in pulmonary and oncologic imaging. In our study, the ability to segment normally aerated, infiltrated, and collapsed lung regions using standard HU thresholds (−1050 to 0 HU) enabled detailed quantification of both total and affected lung volumes.

The platform also facilitates reproducible workflows, allows custom thresholding aligned with current radiologic standards, and integrates seamlessly with DICOM datasets, making it well-suited for both retrospective and prospective volumetric studies. As commercial solutions may be cost-prohibitive or limited in academic and low-resource settings, 3D Slicer represents a robust and validated alternative for lung volumetry in research and potentially clinical practice [15,16,17,18]

Our study revealed significant lung volume loss during the acute phase, driven by alveolar collapse, infiltrates, and fibrosis, with partial recovery observed over time. These results align with findings from Lin et al. (2021) [19], who emphasized the utility of CT volumetry for quantifying COVID-19-associated lung damage.

The histopathological findings in this study, including marked alveolar area reduction and early signs of interstitial fibrosis, align with prior reports on COVID-19-induced lung injury. Notably, Chrabańska et al. (2022) [20] described epithelial cell dysfunction, alveolar–capillary barrier disruption, and extracellular matrix remodeling as characteristic features of viral pneumonia and acute respiratory distress syndrome (ARDS). These parallels support the broader relevance of our observations and reinforce the structural basis of the radiological volume loss seen on CT. While the underlying mechanisms are consistent, the methodological approach used in this study offers added value. By employing quantitative digital microscopy, we were able to directly measure alveolar surface area in both preserved and infiltrated regions, moving beyond traditional histologic description. This allowed for objective quantification, showing a greater than 65% reduction in the alveolar area in affected zones. Such data provide high-resolution structural evidence that the regions identified as infiltrated or collapsed on CT correspond to the anatomically disrupted and functionally compromised lung tissue.

These results strengthen the pathophysiological interpretation of radiological findings and support the use of combined radiological–histological analysis in understanding severe viral pneumonia.

Our findings are not limited to COVID-19, as similar histopathological patterns in other viral pneumonias, such as influenza, have been described, suggesting that the mechanisms of alveolar damage, surfactant dysfunction, and fibrosis observed in COVID-19 may be extrapolated to other types of respiratory infections.

Although this study focused on COVID-19 pneumonia, the structural and histopathological patterns observed are not exclusive to SARS-CoV-2. Similar mechanisms of alveolar damage, surfactant dysfunction, and early fibrotic remodeling have been described in other respiratory infections. These shared features suggest that the volumetric and histological changes quantified in this study likely reflect a broader response pattern to severe viral pneumonia.

This study has several limitations that must be considered when interpreting the results. First, the relatively small sample size of 43 patients limits its statistical significance and may affect the generalizability of the findings. Although the observed correlations were strong and statistically significant, confirmation in larger, multicenter cohorts is necessary to validate these observations and extend their applicability to broader patient populations. Second, the retrospective nature of the study introduces the possibility of selection bias, as only patients with available high-resolution CT and follow-up imaging were included.

## 5. Conclusions

Monitoring patients with moderate and severe forms of COVID-19 has revealed a significant increase in lung volumes following the resolution of the acute phase of the illness. This ongoing observation has provided valuable insights into the mechanisms of respiratory failure induced by the SARS-CoV-2 virus. This study demonstrates that quantitative CT volumetry, combined with histopathological validation, provides a robust and objective method for assessing lung volume loss in patients with moderate to severe COVID-19 pneumonia. A strong positive correlation was found between the percentage of affected lung parenchyma (−650 to 0 HU) and the percentage of total lung volume reduction (r = 0.72, *p* < 0.000001), supporting the structural-functional relevance of radiological findings.

Patients with more than 25% affected lung volume exhibited significantly greater median volume reduction (1730.4 mL and 1993.4 mL) compared to those with <25% (284.4 mL, *p* < 0.0001, Kruskal–Wallis test), highlighting the value of HU-based classification over semi-quantitative CT severity scoring, which showed less consistent discrimination between groups after Bonferroni correction. Volumetric analysis was performed using 3D Slicer, an open-source platform that yielded reproducible results across patients and disease stages. Histological evaluation of severe cases confirmed a >65% reduction in the alveolar area within radiologically affected zones, establishing a direct anatomical correlate to the loss of ventilated lung volume observed on CT.

These findings illustrate that lung volume loss in acute viral pneumonia is both quantifiable and clinically meaningful, with implications not only for COVID-19 but for understanding the structural basis of respiratory failure in other forms of severe viral lung injury.

## Figures and Tables

**Table 1:** Imaging-based severity CT scores and quantitative metrics.

Case Id	Sex	Lung Volume Acute_Phase (cm^3^)	Lung Volume Follow Up (cm^3^)	Volume Recovered	Precent Volume Changes	Volume Infiltrate (−650 to −200)	Percent Infiltrate Volume	Volume Collapsed (−200 HU to 0 HU)	Volume Affected (Infiltrate + Collapsed)	Percent Affected (Infiltrate + Collapsed	CT Score
1	m	3492.01	5445.44	1953.43	35.87	835.96	23.94	244.38	1080.35	30.94	20
2	m	3478.02	5582.96	2104.94	37.70	1313.91	37.78	272.36	1586.27	45.61	12
3	f	5868.00	5967.20	99.20	1.66	323.91	5.52	32.24	356.15	6.07	7
4	m	3941.58	6742.76	2801.18	41.54	1190.18	30.20	290.47	1480.64	37.56	13
5	m	6078.97	6945.56	866.59	12.48	440.22	7.24	43.60	483.82	7.96	10
6	m	2769.81	6773.85	4004.04	59.11	1280.76	46.24	215.64	1496.40	54.03	14
7	m	6363.99	7478.45	1114.46	14.90	1036.25	16.28	73.89	1110.14	17.44	15
8	m	2285.92	3946.93	1661.01	42.08	1345.96	58.88	472.47	1818.42	79.55	23
9	f	2607.59	3265.51	657.92	20.15	741.92	28.45	178.79	920.71	35.31	15
10	m	3392.49	5361.41	1968.92	36.72	1831.59	53.99	223.11	1977.51	58.29	20
11	m	4347.87	6265.75	1917.88	30.61	1831.59	42.13	312.22	2143.81	49.31	23
12	f	1408.75	2541.38	1132.63	44.57	764.48	54.27	292.60	1057.08	75.04	20
13	f	3622.98	4915.54	1292.56	26.30	992.66	27.40	169.37	1162.03	32.07	17
14	m	2977.80	4996.62	2018.82	40.40	948.42	31.85	314.42	1262.84	42.41	12
15	m	2742.08	4390.05	1647.97	37.54	1187.42	43.30	346.05	1533.47	55.92	16
16	m	2908.46	4269.87	1361.41	31.88	923.16	31.74	539.56	1462.71	50.29	13
17	m	3900.00	6234.59	2334.59	37.45	1937.49	49.68	761.98	2699.47	69.22	17
18	m	4059.14	7492.26	3433.12	45.82	1831.59	45.12	563.81	2395.40	59.01	23
19	m	5947.84	7063.01	1115.17	15.79	579.79	9.75	55.16	634.95	10.68	13
20	m	6111.11	5875.82	−235.29	−4.00	721.38	11.80	57.98	779.36	12.75	15
21	f	2072.40	4153.02	2080.62	50.10	1133.48	54.69	233.04	1366.52	65.94	20
22	f	3343.55	3501.26	157.71	4.50	781.66	23.38	151.30	932.96	27.90	11
23	m	4266.39	5732.32	1465.93	25.57	1297.27	30.41	137.15	1434.42	33.62	20
24	m	3431.16	4560.25	1129.09	24.76	559.59	16.31	559.59	906.50	26.42	15
25	f	3626.36	3759.05	132.69	3.53	396.51	10.93	29.66	426.17	11.75	13
26	m	6523.16	7162.46	639.30	8.93	2729.35	41.84	739.93	3469.28	53.18	18
27	m	5004.04	7021.82	2017.78	28.74	2489.47	49.75	309.22	2798.69	55.93	17
28	f	4060.20	4344.55	284.35	6.54	394.63	9.72	37.44	432.08	10.64	5
29	m	5709.18	5821.87	112.69	1.94	676.68	11.85	63.98	740.67	12.97	7
30	m	2870.78	5055.50	2184.72	43.21	1478.66	51.51	292.10	1770.75	61.68	20
31	f	2403.28	4642.14	2238.86	48.23	1323.14	55.06	494.65	1817.79	75.64	20
32	m	3142.77	5829.58	2686.81	46.09	1214.54	38.65	110.44	1324.98	42.16	20
33	m	4546.81	6341.85	1795.04	28.30	541.90	11.92	31.67	573.57	12.61	16
34	m	3218.36	5560.63	2342.27	42.12	1084.22	33.69	82.09	1324.10	41.14	6
35	m	5840.16	5945.49	105.33	1.77	640.43	10.97	55.89	696.17	11.92	15
36	m	2954.57	4999.31	2044.74	40.90	1011.42	34.23	229.38	1241.16	42.01	12
37	f	3947.82	4528.98	581.16	12.83	457.58	11.59	229.39	486.59	12.33	5
38	m	3035.65	4992.96	1957.31	39.20	1114.79	36.72	183.96	1298.75	42.78	18
39	f	2809.27	4352.24	1542.97	35.45	665.74	23.70	46.99	712.73	25.37	13
40	f	3137.57	4227.28	1089.71	25.78	1788.54	57.00	285.92	2074.45	66.12	11
41	f	1778.58	3154.44	1375.86	43.62	808.95	45.48	78.84	887.79	49.92	17
42	m	3399.02	3408.76	9.74	0.29	1319.39	38.82	164.81	1484.20	43.67	16
43	f	2714.58	3476.02	761.44	21.91	878.75	32.37	83.48	962.23	35.45	12

**Table 2:** Normal alveolar area vs. infiltrated area.

	Case 1	Case 2	Case 3
Normal	706,205 µm^2^	581,262 µm^2^	623,296 µm^2^
Infiltrated	216,230 µm^2^	47,389 µm^2^	217,976 µm^2^
Alveolar volume loss	489,975 µm^2^	533,873 µm^2^	405,320 µm^2^
% loss	69.38%	91.85%	65.03%

## Data Availability

The data presented in this study are available upon reasonable request from the corresponding author.
